# Metabolic reprogramming in cancer cells: glycolysis, glutaminolysis, and Bcl-2 proteins as novel therapeutic targets for cancer

**DOI:** 10.1186/s12957-016-0769-9

**Published:** 2016-01-20

**Authors:** Chunxia Li, Guifeng Zhang, Lei Zhao, Zhijun Ma, Hongbing Chen

**Affiliations:** 1 Department of Oncology, Yidu Central Hospital, Weifang, Shandong 262500 China; 2 Department of Oncology Nursing, Yidu Central Hospital, Weifang, Shandong 262500 China; 3 Department of Pediatrics, Yidu Central Hospital, Weifang, Shandong 262500 China

## Abstract

Nearly a century ago, Otto Warburg made the ground-breaking observation that cancer cells, unlike normal cells, prefer a seemingly inefficient mechanism of glucose metabolism: aerobic glycolysis, a phenomenon now referred to as the Warburg effect. The finding that rapidly proliferating cancer cells favors incomplete metabolism of glucose, producing large amounts of lactate as opposed to synthesizing ATP to sustain cell growth, has confounded scientists for years. Further investigation into the metabolic phenotype of cancer has expanded our understanding of this puzzling conundrum, and has opened new avenues for the development of anti-cancer therapies. Enhanced glycolytic flux is now known to allow for increased synthesis of intermediates for sustaining anabolic pathways critical for cancer cell growth. Alongside the increase in glycolysis, cancer cells transform their mitochondria into synthesis machines supported by augmented glutaminolysis, supplying lipid production, amino acid synthesis, and the pentose phosphate pathways. Inhibition of several of the key enzymes involved in these pathways has been demonstrated to effectively obstruct cancer cell growth and multiplication, sensitizing them to apoptosis. The modulation of various regulatory proteins involved in metabolic processes is central to cancerous reprogramming of metabolism. The finding that members of one of the major protein families involved in cell death regulation also aberrantly regulated in cancers, the Bcl-2 family of proteins, are also critical mediators of metabolic pathways, provides strong evidence for the importance of the metabolic shift to cancer cell survival. Targeting the anti-apoptotic members of the Bcl-2 family of proteins is proving to be a successful way to selectively target cancer cells and induce apoptosis. Further understanding of how cancer cells modify metabolic regulation to increase channeling of substrates into biosynthesis will allow for the discovery of novel drug targets to treat cancer. In the present review, we focused on the recent developments in therapeutic targeting of different steps in glycolysis, glutaminolysis and on the metabolic regulatory role of Bcl-2 family proteins.

## Background

In 1924, Otto Warburg made the landmark discovery that, unlike most somatic cells that rely heavily upon oxidative phosphorylation for efficient and adequate synthesis of ATP to sustain their functions, cancer cells predominantly depend on aerobic glycolysis and produce large amounts of lactate [1]. Why cancer cells would favor a less efficient mechanism for energy production has long puzzled researchers—a rapidly proliferating cell would be expected to have extensive energy demands. Initially, it was surmised that this metabolic switch resulted from damage to mitochondrial function. For nearly a century since the first observations of the “Warburg effect”, increasing evidence has led to the realization that the metabolic switch in cancer cells is in fact the result of a highly complex, insidious process of reprogramming achieving a metabolic state ideal for the proliferation and sustenance of cancer cells. Historically, mutations in genes regulating proliferation and growth signaling were thought to be the primary triggers of carcinogenesis, changes in metabolism seen as the cells simply “keeping up” with the demands of higher multiplication rates. However, the finding that many of these oncogenes moonlight as critical regulators of metabolism and that their dysregulation contributes to altered metabolic phenotypes that favor growth, has called for a re-evaluation of metabolic reprogramming as an emerging hallmark of cancer [2, 3]. A significant shift in our understanding of metabolic state as a central transformative force in cancer cell development was sparked by observation that changes in metabolism can modulate a cell’s ability to differentiate [4, 5], diverging from the paradigm that metabolic reprogramming is an adaptation to mutations in order to maintain biosynthesis and suggesting that an altered metabolic state can itself enhance growth and survival. Continued research in this area may lead to the development of powerful therapeutics that can selectively target cancer cells by obstructing their metabolic evolution. The reprogrammed metabolism of cancer cells is instrumental in achieving other well-described hallmarks of cancer such as limitless proliferation and escape from apoptosis. Many of the metabolites (e.g., (R)-2-hydroxybutyrate, lactate, etc.) that are specifically elevated in cancer cells promote not only their proliferation and survival but also prevent their apoptosis, by either activating the anti-apoptotic Bcl-2 family proteins or increasing their expression.

In this review, we addressed the recent developments in our understanding on the derangements in cancer cell glycolysis and glutaminolysis and on the role of Bcl-2 family proteins including Mcl-1, NOXA, Bad, etc., in the regulation of reprogrammed metabolism in cancer cells. We highlighted the various suggested novel anti-cancer therapeutic targets in these metabolic pathways.

### The fates of glucose in cancer cell metabolism

**Fig. 1:**
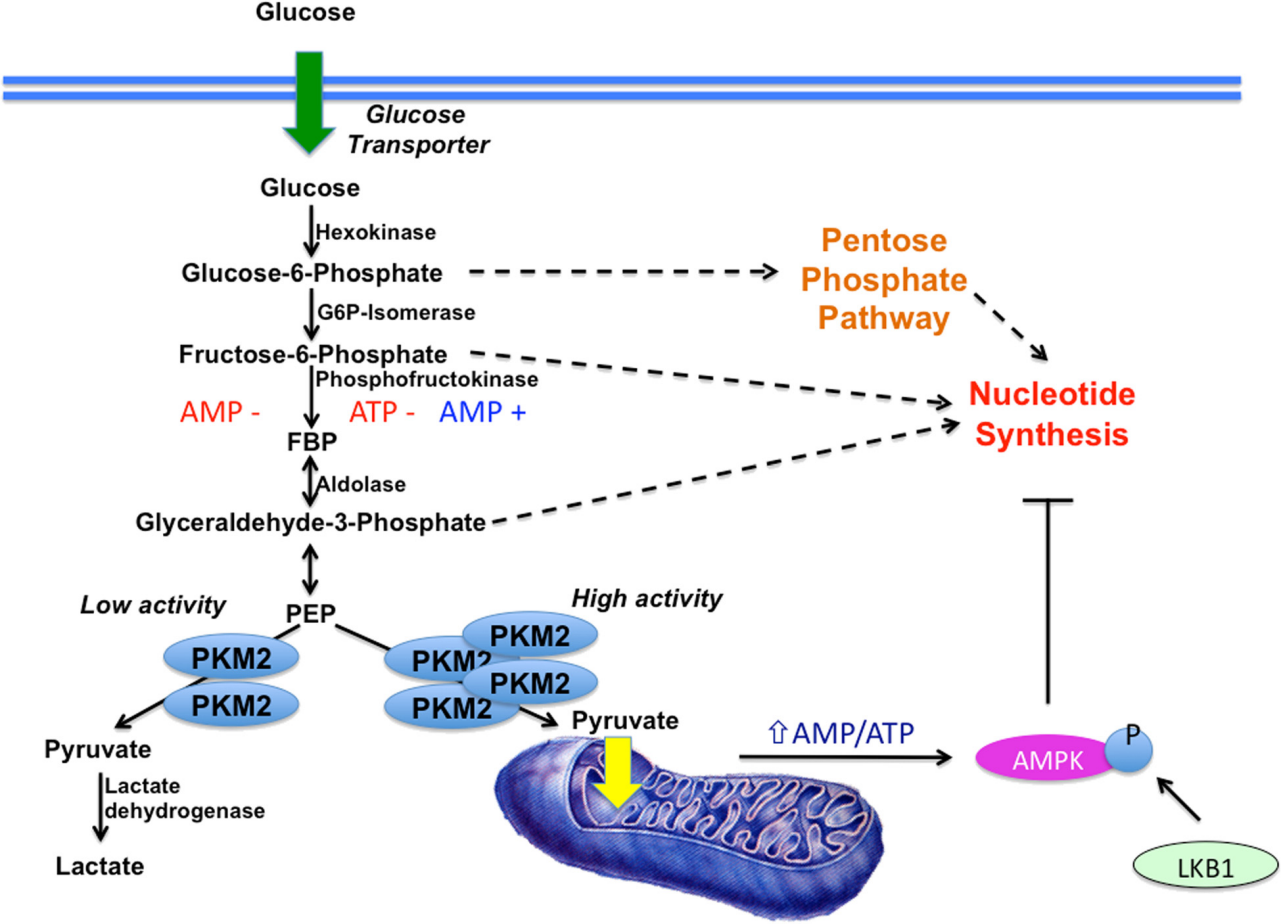
Reprogramming of the glycolytic pathway in cancer cells. Cancer cells rely on increased glycolytic flux, increasing glucose uptake, and producing large amounts of lactate. The expression of the PKM2 isoform allows for flexibility in that the dimeric form will exhibit decreased activity, promoting accumulation of glycolytic intermediates that can be shunted into the pentose phosphate pathway and nucleotide synthesis. Decreased oxidative phosphorylation in normal cells would lead to decreased energy charge and increased AMP, activating AMPK. Cancer cells often harbor mutations in the upstream AMPK kinase LKB1, without whose phosphorylation activity, AMPK cannot be activated and therefore cannot inhibit anabolic pathways such as nucleotide synthesis

When completely oxidized, 1 glucose can provide up to 36 adenosine triphosphate (ATP) molecules, compared to a meager 2 ATPs per glucose via conversion to lactate through glycolysis [6]. To reconcile the befuddling discovery that cancer cells sustain rapid proliferative rates using the comparatively inefficient process of aerobic glycolysis, Warburg hypothesized that cancer cells may have defective mitochondria incapable of maintaining oxidative respiration [7]. However, it has been demonstrated that cancer cells do not necessarily exhibit compromised mitochondrial function [8, 9], suggesting that another mechanism underlies this metabolic phenotype. Aerobic glycolysis is indeed sufficient to fuel rapid proliferation, as seen in the case of many unicellular organisms such as yeast [10, 11]. Cancer cells favoring glycolysis maintain energy charge, as determined by the ratio of ATP:ADP and NAD(P)H:NAD(P)+ (NAD is nicotinamide adenine dinucleotide; NADP is nicotinamide adenine dinucleotide phosphate), suggesting that energy depletion does not result as a consequence of this metabolic shift [12, 13]. This could be due to the fact that cancer cells do not experience substrate scarcity, and are continually supplied with glucose and other nutrients from the blood. Additionally, normal cells tightly regulate energy charge and ATP synthesis to prevent uncontrolled proliferation even in the presence of abundant resources (Fig. 1). For example, increases in ATP will negatively feedback on the glycolytic pathway by inhibiting phosphofructokinase (PFK) and pyruvate kinase (PK), decreasing the conversion of glucose to pyruvate and lactate [14, 15]. When ATP is depleted, increases in AMP will activate AMPK, leading to the inhibition of anabolic processes such as protein synthesis and triggering catabolism, such as fatty acid oxidation, to replenish ATP [16]. Precise detection of cellular energy state allows for fine-tuned regulation of anabolic and catabolic pathways in the cell—interestingly, many cancer cell types show mutations in the upstream AMPK kinase LKB1, which is required for phosphorylation and activation of AMPK [17]. LKB1 is considered to be a tumor suppressor, and the loss of its function may contribute to carcinogenesis by eliminating the AMPK energy sensor and dissociating the balance between energy state and biosynthesis [18, 19]. It has been demonstrated that LKB1-deficient cancers are susceptible to treatment with the biguanide phenformin, phenformin selectively inducing apoptosis in these cancer cells as opposed to cancer cell types harboring other mutations [20]. These findings suggest that there is potential for the use of phenformin, and perhaps related compounds, in the treatment of those cancers with LKB1 defects. Activation of AMPK may also prove to be a promising target for the development of novel therapeutics against cancer. Cell culture studies have shown that activation of AMPK in cancer cells sensitizes them to chemotherapeutic agents currently used in patients [21, 22]. AMPK activators could be developed into powerful therapeutics that could be used in combination with other drugs to more effectively treat cancer (Fig. 1).

It has also been found that many cancer cell types preferentially express an isoform of PK, PKM2. Unlike other isoforms of this enzyme that are consistently in a tetrameric state and are largely regulated by allosteric inhibition by ATP and activation by PEP and fructose 1,6-bisphosphate, PKM2 can exist in either a highly active tetrameric state or a less active dimeric state. The PKM2 isoform is expressed in fetal tissues, but is eventually replaced with other isoforms later in development [23]. PKM2 offers tumor cells the advantage of increased metabolic flexibility—this enzyme is chiefly retained in its dimeric state in cancer cells, allowing glycolytic intermediates to build-up and channel into anabolic pathways such as phospholipid, nucleotide, and amino acid synthesis that can sustain cellular replication [12, 24]. If necessary, however, PKM2 can also switch to its active tetrameric form in response to modulation by fructose 1,6-bisphosphate in order to increase pyruvate synthesis and increase ATP production. Studies investigating the possibility of activating PKM2 to hamper the capacity of cancer cells to accrue crucial biosynthetic intermediates have provided evidence that the use of small-molecule PKM2 activators may indeed be effective in impeding tumor growth [25, 26].

As mentioned above, cancer cells, which conduct high levels of glycolysis, produce lactate in large amounts and this metabolite is transported out of the cell into extracellular environment. Glycolytically produced pyruvate is converted to lactate by lactate dehydrogenase (LDH), and this step is essential for the regeneration of NAD^+^ in the cytosol to let high rates of glycolysis prevail, which is needed for oncogenesis. There are five different LDH subtypes (LDH 1–5), which occur as homo- or hetero-tetramers of muscle-type subunits encoded by LDH-A gene and heart-type subunits encoded by LDH-B gene. Cellular levels of lactate are governed by differential expression of LDH isoforms, the lactate monocarboxylate transporter (MCT), and the oxidative capacity of cell. Many studies have shown that several types of cancer cells require LDH-A gene, which encodes LDH-5 isoenzyme, for their maintenance and proliferation [27, 28], even though the precise mechanisms by which LDH-5 promotes cancer progression is not known. LDH-5 expression by LDH-A gene is under the control of HIF-1α and is elevated under hypoxic conditions. In fact, it is well known that HIF-1α expression is elevated in many cancers and is known to offer protection to cancer cells. Expression of HIF-1α and LDH-5 are found to be correlated and associated with poor prognosis of many cancers [29]. Importance of LDH-5 as an anti-cancer target has been explored extensively, and it has been found that either suppression of LDH-5 levels by RNAi-based approaches or by pharmacological inhibition cause significantly attenuated cancer cell proliferation and apoptosis [27, 30]. Myc oncogene, which is activated in many cancers, is known to regulate the transcription of several cellular proliferation-related genes and microRNAs and also enhances the expression of LDH-A [31]. Many cancer cells convert significant proportion of glucose to lactate, thus producing an acidic microenvironment surrounding the cancerous tissue. This acidification of the extracellular microenvironment is known to be advantageous to the tumors, by favoring invasion and suppression of cytotoxic T lymphocytes. Transport of lactate along with a proton, out of the cell is facilitated by MCT and there are many isoforms of MCT. It has been shown that inhibition of MCT by pharmacological agents was effective in curtailing angiogenesis and tumor progression of gliomas [32]. It has been suggested recently that MCTs, which are overexpressed in cancers act as “Trojan horses” as they can be exploited for transporting bromopyruvate, an anti-cancer agent into cancer cells to inhibit glycolysis [33]. Thus, overall, it appears that glycolysis-derived lactate and its handling by LDH and MCT in cancer cells provide growth advantage.

### Glutaminolysis: the mitochondria as an anabolic apparatus

**Fig. 2:**
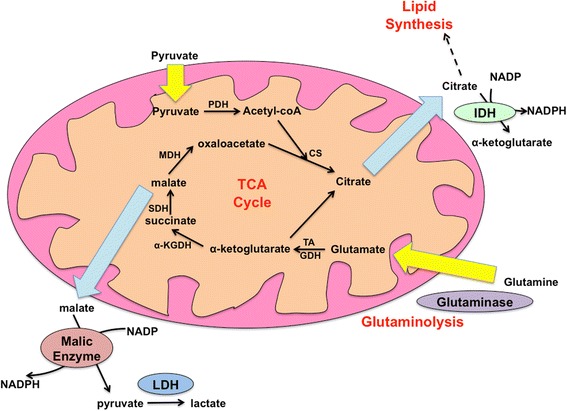
Increased glutaminolysis and the role of the mitochondria in cancer cells. The mitochondria of cancer cells switch from the canonical function of oxidative phosphorylation and ATP production to synthesis of anabolic intermediates that can be utilized for lipid and amino acid synthesis. This is supported by high rates of glutaminolysis, Glutamine is converted to glutamate by glutaminase, and glutamate is then transaminated into α-ketoglutarate, which contributes to citrate and malate synthesis. Both of these metabolites can then be exported from the mitochondria, malate converted to pyruvate and then lactate to produce NADPH. Citrate can also be metabolized to α-ketoglutarate, synthesizing NADPH, or alternatively be channeled into lipid synthesis. *PDH* pyruvate dehydrogenase complex, *MDH* malate dehydrogenase, *SDH* succinate dehydrogenase, *α*-*KGDH* α-ketoglutarate dehydrogenase complex, *CS* citrate synthase, *TA* transaminase, *GDC* glutamate dehydrogenase

Apart from glucose, the other major substrate that contributes to energy production is glutamine [34]. Glutaminolysis is upregulated in many types of cancer, glutamine providing the crucial source of nitrogen to these rapidly proliferating cells for amino acid synthesis via glutamate production and transamination. Furthermore, instead of oxidizing glutamine completely to produce ATP, the mitochondria of cancer cells shunt glutamine into citrate for lipid production and for the production of NADPH by isocitrate dehydrogenase (IDH), and into malate which can also be converted to pyruvate by malic enzyme and produce NADPH [35]. Hence, both glucose and glutamine are utilized to replenish synthetic intermediates as opposed to being oxidized for ATP—though it may appear that this form of metabolism is inefficient, the increased production of ATP does not meet the requirements of a rapidly multiplying cell as effectively. As a result of this switch to increased glutamine dependence by the mitochondria, the diminished contribution of glucose to the tricarboxylic acid (TCA) cycle is well-compensated for by contributions from glutamine, and the mitochondria are transformed into apparatuses for the synthesis of building blocks required to sustain rapid cell division (Fig. 2). This mitochondrial reprogramming results from increased expression of the oncogenic transcription factor Myc, which increases expression of glutaminase and glutamine transporters to support increased glutaminolysis, and also augments expression of lactate dehydrogenase A (LDH-A) which allows shunting of glucose-derived pyruvate away from the mitochondria and into lactate [31, 36].

This glutamine “addiction” observed in cancer cells suggests that targeting any of the pathways involved in maintaining glutamine metabolism would exhaust cancer cells of a key substrate supply. Since increased NADPH production is one of the major benefits of this altered glutamine metabolism, a likely candidate for novel drug design is IDH. Furthermore, IDH activity is important to maintain redox regulation and scavenge reactive oxygen species (ROS) that may otherwise cause extensive damage and trigger apoptosis. Cancer cells with ablated expression of the mitochondrial isoform IDH2, had diminished capacity to form tumors when grafted into mice as compared with cancer cells where IDH2 expression was retained [37]. Interestingly, in many cancers such as glioma, colorectal cancer, and acute myeloid leukemia, IDH is mutated such that its activity is enhanced and it produces an “oncometabolite” called (R)-2-hydroxyglutarate from α-ketoglutarate. This mutation allows for increased flux of glutamine into lipid production, and (R)-2-hydroxyglutarate itself is thought to inhibit oxidative phosphorylation by inhibiting complexes of the electron transport chain, favoring channeling of glutamine away from oxidation and towards anabolic pathways [38, 39]. Additionally, mutated IDH was found to inhibit the expression of HNF4α, an important regulator of hepatocyte differentiation and proliferation, allowing liver progenitor cells to evade differentiation and maintain high replicative rates [40]. Targeting mutant IDH and decreasing its activity can effectively impose differentiation upon cancer cells, limiting their replicative potential and rendering them more susceptible to drugs that induce apoptosis [41]. Finally, tumors harboring IDH mutations are especially reliant on glutamine metabolism—another promising drug target is glutaminase, whose inhibition would starve cancer cells of glutamine, essentially shutting down biosynthesis [42, 43].

### The dual personalities of the Bcl-2 family proteins

Some of the most groundbreaking discoveries highlighting the importance of metabolic reprogramming in cancer development were those implicating members of the Bcl-2 family of proteins as metabolic regulators. Bcl-2 family proteins have long been recognized as crucial mediators of mitochondrial apoptotic signaling, also referred to as intrinsic apoptosis [44, 45]. These proteins share close homologous domains known as Bcl-2 homology (BH) domains—several of the multi-domain members may contain 3–4 BH domains, Mcl-1, Bcl-2, and Bcl-xL being some of the anti-apoptotic proteins of this variety and Bak and Bax the pro-apoptotic. Some of these proteins also contain transmembrane domains, allowing them to directly embed into the mitochondrial and endoplasmic reticulum membranes [46]. Several pro-apoptotic single domain proteins, the BH3-only proteins, contain only the BH3 domain considered to be the minimal death domain, and play significant roles in determining the balance between pro- and anti-apoptotic signaling [47]. The specific interactions amongst different Bcl-2 proteins are what ultimately decide whether the intrinsic cell death pathway is activated. The pro-apoptotic Bak and Bax, both transmembrane proteins, can oligomerize to form pores, or mitochondrial outer membrane permeabilization (MOMP) that allows release of mitochondrial cytochrome c into the cytoplasm, triggering apoptosis [48, 49]. The anti-apoptotic Bcl-2 proteins do not form oligomers, and can complex with Bak or Bax preventing their oligomerization. Some of the BH3-only proteins will bind anti-apoptotic Bcl-2 proteins, sequester them away from Bax/Bak and in this way negate their anti-apoptotic effect. Other BH3-only proteins can interact with Bak and Bax, promoting their oligomerization [50].

Anti-apoptotic Bcl-2 family proteins such as Bcl-2, Bcl-xL, and Mcl-1 are frequently upregulated in various forms of cancer, promoting tumorigenesis and resistance to chemotherapy. In recent years, significant progress has been made in the development of a novel class of anti-cancer drugs that specifically inhibit the anti-apoptotic Bcl-2 family proteins, or act as BH3 mimetics. These drugs have a critical advantage over classical cytotoxic therapies that do not possess the same degree of specificity for cancerous cells and have many toxic side effects [51–53]. The fascinating discovery that many of the Bcl-2 family proteins are also the key modulators of metabolism, and that their role as apoptotic mediators is in fact a secondary function, suggests that cells naturally link dysregulation of metabolism or the unavailability of substrates to cell death, a process that involves nutrient dependent changes in the expression and activity of pro- (PUMA, NOXA, Bad, and Bim) and anti-(Mcl-1) apoptotic proteins. It is also possible that cancer cell reprogramming involves altering Bcl-2 proteins to sustain a metabolic phenotype suitable for increased growth. The earliest confirmation of a metabolic role for Bcl-2 proteins was the finding that BH3-only protein BAD resides in a complex with glucokinase [54]. Glucokinase is crucial for detection of glucose in hepatocytes and pancreatic B cells, and due to its high Km for glucose and absence of product feedback inhibition, the glucokinase reaction is entirely substrate driven and acts as an ideal substrate sensor. BAD will activate glucokinase via direct interaction, which is promoted by phosphorylation of BAD by kinases such as Akt. When bound to glucokinase, BAD’s pro-apoptotic functions are defused. However, dephosphorylated BAD will dissociate from glucokinase, freeing it to bind with anti-apoptotic Bcl-2 or Bcl-xL and promote apoptosis. In certain types of cancers, it has been demonstrated that BAD phosphorylation is increased due to the increased activities of upstream kinases, and that inhibition of BAD phosphorylation decreases cancer cell survival [55–57]. Sequestration of BAD to the glucokinase complex will not only prevent its pro-apoptotic functions, but will also promote increased glucokinase activity and glycolysis, a key metabolic shift that favors biosynthesis and proliferation. Although dephosphorylation of BAD alone is not adequate to trigger apoptosis, it shifts the balance towards cell death and uncouples the favorable metabolic signal of high glucose flux from regulation of cell survival.

Besides the modification of expression and activity of Bcl-2 family proteins, metabolic reprogramming in cancer cells ensures the supply of necessary metabolite building blocks for cell proliferation. Thus, the elevated glycolysis ensures the supply of glycolytic intermediates glycerol-3-phosphate and dihydroxyacetone phosphate, needed for the biosynthesis of phospholipids, the major constituents of cell membranes. Similarly, reprogramming of lipid metabolism ensures the synthesis of fatty acids and other lipids, needed for membranogenesis. In fact, the upregulated glutaminolysis not only supports the energy needs of the cancer cells but also provides the needed precursors for lipogenesis via citrate cycle, as mentioned above in the glutaminolysis section.

Another BH3-only member of the Bcl-2 family involved in metabolic regulation is NOXA. The cyclin-dependant kinase 5 (CDK5) will phosphorylate NOXA in the presence of high glucose. Phosphorylated NOXA localizes to the cytoplasm and is incapable of executing its pro-apoptotic functions—instead, it complexes with Mcl-1L and promotes increased glucose metabolism and augments flux through the pentose phosphate pathway, supporting nucleotide synthesis [58]. Indeed, NOXA is constitutively expressed in cancer cells, and many cancers exhibit increased CDK5 activity that is important for tumor growth and survival of thyroid and neuroendocrine cancers [59, 60]. Modification of the metabolic and apoptotic roles of Bcl-2 family proteins is one of the major mechanisms by which cancer cells achieve the metabolic shift necessary to meet the growth requirements. The effectiveness of therapeutics targeting this family of proteins suggests that drugs that can both increase cancer cell susceptibility to apoptosis and undo tumorigenic metabolic reprogramming may prove to be much more potent in treating cancer.

## Conclusions

A consistent feature of tumor metabolism is increased glycolysis and glutaminolysis, both of which are used to supply the intermediates required to sustain amino acid synthesis, the pentose phosphate pathway, and lipid production. These metabolic outcomes are achieved via several mechanisms—however, strategies targeting cancer cell metabolism and inhibiting the Warburg effect are showing great promise as powerful, novel therapies to target cancer. Nearly a century after the first observations that cancer cells maintain altered metabolic features, the significance of metabolic reprogramming in carcinogenesis and its relevance to anti-cancer drug design continues to grow. Further research in this field will shed light on tumor progression, and how to most effectively “starve” cancer.
